# Sleep Disordered Breathing and Recurrent Tonsillitis Are Associated With Polymicrobial Bacterial Biofilm Infections Suggesting a Role for Anti-Biofilm Therapies

**DOI:** 10.3389/fcimb.2022.831887

**Published:** 2022-02-28

**Authors:** Tulia Mateus, Elke J. Seppanen, Camilla de Gier, Sharon Clark, Harvey Coates, Shyan Vijayasekaran, Karen Prosser, Selma P. Wiertsema, Angela Fuery, Lea-Ann S. Kirkham, Peter C. Richmond, Ruth B. Thornton

**Affiliations:** ^1^ School of Medicine, University of Western Australia, Perth, WA, Australia; ^2^ Wesfarmers Centre of Vaccines and Infectious Diseases, Telethon Kids Institute, Perth, WA, Australia; ^3^ Perth Children’s Hospital, Perth, WA, Australia; ^4^ Centre for Child Health Research, University of Western Australia, Perth, WA, Australia

**Keywords:** pediatric sleep disordered breathing, recurrent tonsillitis, nontypeable *Haemophilus influenzae*, *Streptococcus pneumoniae*, *Streptococcus pyogenes*, bacteria, biofilms

## Abstract

**Background:**

The underlying pathogenesis of pediatric obstructive sleep disordered breathing (SDB) and recurrent tonsillitis (RT) are poorly understood but need to be elucidated to develop less invasive treatment and prevention strategies.

**Methods:**

Children aged between 1- and 16-years undergoing adenoidectomy, tonsillectomy or adenotonsillectomy for SDB (n=40), RT alone (n=18), or both SDB and RT (SDB+RT) (n=17) were recruited with age-matched healthy controls (n=33). Total bacterial load and species-specific densities of nontypeable *Haemophilus influenzae* (NTHi)*, Staphylococcus aureus, Streptococcus pyogenes, Streptococcus pneumoniae* and *Moraxella catarrhalis* were measured by qPCR in nasopharyngeal swabs, oropharyngeal swabs, adenoid and tonsillar tissue from children with SDB, SDB+RT and RT, and in naso- and oro- pharyngeal swabs from healthy children. A subset of tonsil biopsies were examined for biofilms using 16S rRNA FISH (n=3/group).

**Results:**

The 5 bacterial species were detected in naso- and oro- pharyngeal samples from all children. These species were frequently detected in adenotonsillar tissue (except *S. aureus*, which was absent in adenoids) from children with SDB, SDB+RT and RT. NTHi and *S. aureus* were observed in tonsils from 66.7-88.2% and 33.3-58.8% of children respectively. Similar total and species-specific bacterial densities were observed in adenotonsillar tissue from children with SDB, SDB+RT or RT. Nasopharyngeal and oropharyngeal swabs were more likely to have multiple bacterial species co-detected than adenotonsillar tissue where one or two targeted species predominated. Polymicrobial biofilms and intracellular bacteria were observed in tonsils from children with adenotonsillar disease.

**Conclusions:**

Antimicrobials, particularly anti-biofilm therapies, may be a strategy for managing children with SDB.

## Introduction

### Adenotonsillectomy Is the Most Common Pediatric Surgery in Australia

Adenotonsillectomy is a common surgical procedure performed on children in high-income countries (Harris et al., 2008). In Australia, it is estimated that ~750 per 100,000 children undergo tonsillectomy (with or without adenoidectomy) each year. This rate has increased by 3% between 2012 and 2018 (Australian Commision on Safety and Quality in Health Care, 2021). Children undergo adenotonsillectomy to treat a range of adenotonsillar diseases associated with infection and/or hypertrophy of the adenotonsillar tissue including sleep disordered breathing (SDB), recurrent tonsillitis (RT), or a combination of both. A definitive diagnosis of obstructive sleep apnoea (OSA) in children with SDB is hampered by the difficulty in obtaining a polysomnogram (PSG). Based on the recommendations most children with a clinical diagnosis of SDB undergo surgery without formal polysomnography (Mitchell et al., 2019). While most children benefit from adenotonsillectomy, with a reduction in primary symptoms and improved quality of life (Marcus et al., 2013), surgery is not without risks nor is it always successful. Post-operative bleeding is the most common complication and accounts for readmissions in ~5% of patients (Ostvoll et al., 2018). Due to long surgical waitlists, many children are left untreated or are managed inefficiently for years before undergoing surgery. Thus, alternative treatment strategies are needed. One potential strategy is to specifically target the pathogen/s or their survival mechanisms that may be associated with adenotonsillar enlargement, and chronic/recurrent infection. Surprisingly, few studies exist comparing the underlying pathogenesis in each of these diseases (Roberts et al., 2012; Viciani et al., 2016; Johnston et al., 2019). Thus, we sought to investigate the presence and densities of common upper respiratory tract bacteria in specimens from children undergoing adenotonsillectomy for SDB, RT or a combination of both. A better understanding of microbial involvement in each of these diseases will facilitate development of alternative or adjunct therapies to surgery.

### Evidence for Microbial Involvement in Sleep Disordered Breathing

While there is considerable evidence for microbial infection driving adenotonsillar enlargement in children with RT (Brodsky et al., 1988; Diaz et al., 2011), a role for microbes in the pathogenesis of SDB is controversial. Recent studies suggest that persistent bacterial infection may drive chronic inflammation, hypertrophy, and airway obstruction in SDB including OSA (Viciani et al., 2016; Johnston et al., 2019; Radcliff et al., 2019), the most severe form of SDB. *Streptococcus pyogenes*-positive throat swabs have been identified in 30% children with OSA compared to 48% of children with RT (Johnston et al., 2019). However, a lack of surface-associated bacterial biofilms on adenoidal and tonsillar surfaces have been shown in children with OSA, while extensive biofilms have been demonstrated in children with RT, and other ear, nose and throat diseases including otitis media and chronic rhinosinusitis (Coticchia et al., 2007; Galli et al., 2007; Zuliani et al., 2009). A recent analysis of bacterial communities from the adenoids and tonsils of children with both RT and SDB revealed that *Proteobacteria* (represented by *Haemophilus*, *Moraxella* and *Neisseria species*) dominated (Johnston et al., 2019). This suggests that these bacteria play a role in adenotonsillar diseases in addition to *S. pyogenes*, which is a well-recognized pathogen associated with RT. Together, these data indicate that the types and densities of bacteria present, and the mechanisms by which they persist in the respiratory tracts and tissues, may contribute to disease pathogenesis and thus, offer targets for new treatment strategies.

### Rationale for Study Design

There is a continuum of diagnoses associated with adenotonsillar disease, with some children diagnosed with RT alone, some having overlapping diagnoses of both SDB and RT, while others are diagnosed with SDB alone. In this study, to represent the continuum of disease and assess associated risk factors, we included children with the aforementioned diagnoses, as well as healthy children with no history of adenotonsillar disease. We evaluated the presence, density, and relative abundance of 5 species of respiratory bacteria that are associated with upper airway and respiratory tissue infections: *S. pyogenes*, *Staphylococcus aureus, Streptococcus pneumoniae*, nontypeable *Haemophilus influenzae* (NTHi) and *Moraxella catarrhalis*, in nasopharyngeal swabs, oropharyngeal swabs, and in adenoid and tonsil tissue from children with adenotonsillar diseases, and in nasopharyngeal and oropharyngeal swabs from healthy controls. We hypothesized that 1) we would detect higher densities of the 5 pathogens in respiratory tissues and swabs from children with RT (or SDB and RT), but lower densities in children with SDB alone who would be more similar to healthy control children; 2) pathogens would be present in polymicrobial biofilms within the tonsils from children with RT but not SDB.

## Methods

### Population

Cases and controls aged between 1 and 16 years were recruited in Perth, Australia between 2005 and 2009. Cases were recruited from the tertiary children’s hospital, Princess Margaret Hospital for Children and controls from the Vaccine Trials Group Database at the Telethon Kids Institute. Data on host and environmental risks factors and antibiotic treatment were collected using a standardized parental questionnaire (Goodwin et al., 2003) and medical records. Written informed consent was obtained from parents or guardians, and assent obtained from children 10 years of age or older prior to any study procedure.

Exclusion criteria for all groups: any chromosomal or craniofacial disorders, known immunodeficiency or receiving immunosuppressive therapy.

Inclusion criteria for Adenotonsillar disease groups: Children were diagnosed as having SDB by the treating otorhinolaryngologist. Due to long waitlists and limited availability of PSG, and in keeping with clinical practice guidelines, the majority of patients with a clinical diagnosis of SDB had surgery without pre-operative PSG. Diagnosis of SDB was based on the history and examination of the child by pediatric otorhinolaryngologists. Specifically, a diagnosis of SDB was assigned if the child had a history of snoring, apneic episodes while sleeping, struggling to breathe, severe dysphagia for solids such as meat, tiredness in the morning, daytime sleepiness, neurocognitive and behavioral issues. Other quality of life symptoms were also considered such as enuresis, night terrors, sleeping in unusual positions, frequent arousals, hypersomnolence, failure to thrive, and obesity. A combination of these findings with the child’s physical examination including consideration of tonsillar size, and any other diagnostic procedures eg radiological or endoscopic examinations. For the purpose of this study, these diagnoses were further supported by the outcomes of the Tucson Children’s Assessment of Sleep Apnoea Study (TuCASA) questionnaire. Of the 13 children that had a PSG conducted in this study, all were confirmed to have OSA. For ease of interpretation, all patients with a clinical diagnosis of SDB or sleep-study proven OSA were analyzed as a single group. Children were diagnosed with RT if they had a history of either 7 tonsillar infections in the preceding 12 months, 5 episodes per year for the previous 24 months, or 3 episodes per year for the previous 36 months (Harris et al., 2008). Two children in the RT group were diagnosed as having chronic tonsillitis exclusively as they had at least one episode of tonsillitis that persisted for 3 or more months as evidenced by daily sore throats, halitosis, cryptic debris, or tonsil stones. Children with concurrent diagnoses of both SDB and RT were grouped into a SDB+RT group. All diagnoses were confirmed by the treating otorhinolaryngologist. Recruitment of children undergoing adenotonsillar surgery was approved by the Princess Margaret Hospital for Children Human Research Ethics Committee, Perth, Australia (1046/EP).

Inclusion criteria for Healthy Controls: Children were considered healthy if they had no significant history of chronic or recurrent respiratory disease including otitis media, lung disease, sinusitis, SDB and recurrent or chronic tonsillitis. Children were recruited through the Vaccine Trials Group at Princess Margaret Hospital for Children, Perth, Australia (1385/EP).

### Specimen Acquisition and Sample Preparation

Nasopharyngeal and oropharyngeal swabs were collected from children with SDB and/or RT at the time of surgery, and from healthy controls when attending the study clinic using flocked swabs (Copan, Italy). Swabs were stored in 1 mL of sterile skim milk tryptone glucose glycerol broth (STGGB), placed on ice and transported to the Princess Margaret Hospital for Children, Children’s Clinical Research Facility laboratory within 4h. Samples were vortexed for 30 sec and stored at −80°C.

Adenoids and tonsils were obtained from children undergoing adenotonsillectomy for SDB and/or RT. Tissues were placed in sterile containers on ice and transported to the Princess Margaret Hospital for Children, Children’s Clinical Research Facility laboratory within 4h of collection. Biopsies, approximately 5mm^3^, were excised from each tonsil and adenoid tissue sample. Biopsies were individually homogenized using hand-held homogenization pestles and placed in cryovials containing 1mL STGGB and stored at −80°C. The remaining tissue was fixed overnight at 4°C in 4% paraformaldehyde, before being washed 3x each for 30min in PBS. They were then stored in 50% PBS/Ethanol at -20°C until sectioning for fluorescence *in situ* hybridization.

### Microbiological Assays

#### Quantitative PCR

Genomic DNA (gDNA) was extracted from nasopharyngeal and oropharyngeal swabs, and from bacterial reference strains NTHi 86-028NP (Bakaletz et al., 1988), *M. catarrhalis* ATCC 25238, *S. pneumoniae* ATCC 7466, *S. pyogenes* ATCC 19615 and *S. aureus* ATCC 25923 using the QIAGEN-DNA Mini Kit (Qiagen, Chadstone, VIC, Australia) as described previously (de Gier et al., 2016). Adenoid and tonsil tissue homogenates were thawed and vortexed for 30 seconds. 200µL of tissue homogenate was used for gDNA extraction. Homogenates were centrifuged at 13000rpm for 7 min to ensure maximum recovery of tissue. The supernatant was discarded, and the pellet resuspended in 180µL of ATL buffer and incubated with 20µL of proteinase K at 56 °C for 3 h. Tissue lysates were loaded into the S-Block from the QIAcube HT Kit (QIAGEN) and gDNA extraction was performed with the QIAcube HT following the manufacturer’s protocol for tissue samples.

Real-time qPCR was conducted using the CFX96 real-time PCR detection system (Bio-Rad, CA, USA) using validated qPCR primers, probes and reaction conditions as described previously (Bhuiyan et al., 2018). Nasopharyngeal and oropharyngeal swab gDNA was diluted 1:20 and adenotonsillar samples, 1:30 in nuclease-free water to reduce assay interference with human DNA. GAPDH qPCR was used on all tissue samples to determine the number of human cells present. The number of bacterial copies were then normalized to the number of human cells to account for variation in biopsy size (Tasker et al., 2010).

#### 16S rRNA FISH

Fluorescence *in situ* hybridization (FISH) was conducted using previously described methods and validated 16S rRNA probes labelled with AlexaFluor 488, 546 or 633 dyes (Invitrogen Technologies, Thornton, NSW) (Hall-Stoodley et al., 2006; Thornton et al., 2011) (**Supplementary Table 1**) to determine persistence mechanisms. A subset of tonsillar biopsies (n=3 children/group) were selected based on a positive qPCR result for at least 1 of the 5 bacterial species tested. 3 FISH probes and 1 nucleic acid stain were used per sample. Slides were mounted using an in-house, low-fade mounting media and imaged using a Nikon A1 confocal laser scanning microscope with 60x oil Plan Apo objectives. Images were analyzed for presence of any bacteria, specific bacterial species, biofilm structure and composition.

Samples were considered positive for biofilm presence when images showed characteristic biofilm morphology and bacterial aggregates resembling microcolonies. Bacterial presence was confirmed by assessment of bacterial morphology based on size (0.5–2 µm), shape (cocci or coccobacilli) and fluorescence in the appropriate channel. Host cells were identified by Hoechst 33342 nucleic acid stain. Bacteria were deemed intracellular when discrete clusters of bacteria were in close proximity to intact host cell nuclei as described previously (Thornton et al., 2011).

### Statistical Analyses

Chi-squared tests were used to compare categorical variables between groups. Kruskal Wallis with *post-hoc* pairwise analyses were used to assess differences in age, episodes of tonsillitis, bacterial species density and total bacterial load between groups. Samples with qPCR measurements below the limit of detection (LOD) were assigned half of this LOD value for statistical analyses. A p value ≤ 0.05 was considered significant. Statistical analyses were performed with SPSS version 23.0 for Windows (SPSS Inc., Chicago, IL, USA) and data were plotted using GraphPad version 8 for Windows (GraphPad Software, La Jolla California USA).

## Results

### Population Characteristics

Forty children were recruited into the SDB group, 17 into the SDB+RT group, 18 into the RT group and 33 into the healthy group. The median age was similar across all 4 groups including healthy controls (p=0.083, **Table 1**). Gender was evenly distributed. Use of antibiotics in the month preceding surgery was higher in children with adenotonsillar diseases compared to healthy controls (p=0.009). Children with RT (with or without concurrent SDB) were more likely to have used antibiotics in the month prior to surgery than children with SDB (RT vs SDB, p=0.015; SDB+RT vs SDB, p=0.024). Only 1 child in the SDB+RT group was on antibiotics at the time of surgery. Although obesity is commonly associated with SDB (Daar et al., 2016), this was not observed in our study with a similar mean body mass index (BMI) observed for each group (means 17.6 to 19.1, p=0.404; **Table 1**). The proportion of children with adenotonsillar diseases presenting with adenotonsillar hypertrophy as determined by grade 3 or 4 adenoids or tonsils on the Brodsky scale (Brodsky et al., 1988) was similar across groups. OSA was confirmed by PSG for 12 of 40 children with SDB and 1 of 17 children with SDB+RT. As PSG could not be obtained for all children diagnosed with SDB, parents were asked to complete the validated Tucson Children’s Assessment of Sleep Apnoea Study (TuCASA) questionnaire (Goodwin et al., 2003) to assess respiratory sleep disturbances. TuCASA responses revealed that healthy children did not exhibit clinical symptoms of respiratory sleep disturbances whereas children diagnosed with an adenotonsillar disease did; witnessed apnoea and snoring were more prevalent in children diagnosed with SDB and SDB+RT compared to RT (p=0.001; p<0.0001 respectively). Learning problems and excessive daytime sleepiness were comparable between children with SDB, SDB+RT and RT. As allergy is also considered as a risk factor for SDB (Gadi et al., 2017; Zheng et al., 2017), we compared allergic presentations across groups. Rhinitis and itchy eyes were more prevalent in children with adenotonsillar diseases compared to healthy controls (p=0.001 and p=0.041) but prevalence amongst children with either SDB, SDB+RT or RT (p=0.625) was similar. The frequency of physician diagnosed hay fever, asthma and eczema/dermatitis were similar across all 4 groups (**Table 1**).

**Table 1:** Study population.

	SDB n = 40	SDB+RT n = 17	RT n = 18	Healthy n = 33	p value
**Median age in years (range)**	6.0 (1.1-13.6)	6.0 (2.3-13.6)	7.8 (2.8-15.8)	9.1 (2.0-16.6)	0.083
**Male Gender n(%)**	22 (55.0)	11 (64.7)	10 (55.6)	13 (39.4)	0.329
**Median no. of acute tonsillitis episodes (range)**	1.0^#^ (0-10)	13.0^#^ (5-45)	20.0^^^ (8-65)	0 (0-3)	0.000
**Procedure**					
- **Adenotosillectomy**	36	16	14	N/A	0.282
- **Tonsillectomy**	2	1	4	N/A	0.097
- **Adenoidectomy**	2	0	0	N/A	0.407
**Mean BMI (+/- SD)^^^**	17.6 (5.2)	17.9 (3.4)	19.1 (3.8)	17.9 (3.3)	0.157
**Antibiotics in last month n(%)**	2^#^ (5.4)	4^#^ (17.6)	5 (27.3)	0^#^	0.009
**Adenoid hypertrophy (Grade 3 or 4) n(%)**	18* (52.9)	9* (64.3)	6* (37.5)	N/A	0.333
**Tonsillar hypertrophy (Grade 3 or 4) n(%)**	22**^#^** (56.4)	12 (70.6)	8 (44.4)	N/A	0.295
**Confirmatory PSG for OSA**	12 (30.0)	1^^^^ (6.7)	0	N/A	–
**TuCASA**					
- **Excessive daytime sleepiness**	16^#^ (41.0)	7^#^ (43.8)	8^**^ (47.1)	1 (3.0)	0.001
- **Witnessed apnoea**	23 (57.5)	9^#^ (56.3)	1^**^ (5.9)	0	0.000
- **Snoring**	36 (90.0)	13^#^ (81.3)	7 (38.9)	0	0.000
- **Learning problems**	10 (25.0)	3^#^ (18.8)	2^**^ (11.8)	0	0.022
**Allergic presentations**					
- **Hay fever**	2 (5.0)	0^#^	1 (5.6)	3 (9.1)	0.629
- **Asthma**	12^#^ (30.8)	3^#^ (18.8)	4 (22.2)	5 (15.6)	0.446
- **Rhinitis**	25 (62.5)	9^^^ (64.3)	7 (38.9)	6 (18.2)	0.001
- **Rhinitis with itchy eyes**	8 (20.0)	5^#^ (31.3)	5 (27.8)	1 (3.0)	0.041
- **Eczema or dermatitis**	11 (27.5)	6^#^ (37.5)	6 (33.3)	7 (21.2)	0.631

SDB, sleep disordered breathing; RT, recurrent tonsillitis; BMI, body mass index; PSG, polysomnogram; OSA, obstructive sleep apnoea; SD, standard deviation.

^^^Information is missing for 4 subjects in SDB group (n = 36), 3 subjects in SDB+RT group (n = 14) and 2 subjects in RT group (n = 16); ^#^information is missing for missing for 1 subject in SDB group (n = 39),1 subject in SDB+RT group (n = 16) and 12 in HC group (n = 21); ^*^Adenoids were not graded from 6 children with SDB (n = 34), 3 children with SDB+RT (n = 14), and 2 children with RT (n = 16); ^^^^Information is missing for 2 subjects in the SDB+RT group (n = 15); ^**^Information is missing for 1 subject in the RT group (n = 17).

N/A, not applicable.

### All Bacterial Species Were Frequently Detected in Nasopharyngeal and Oropharyngeal Swabs From Children With Adenotonsillar Diseases as Well as Healthy Controls

**Figure 1:**
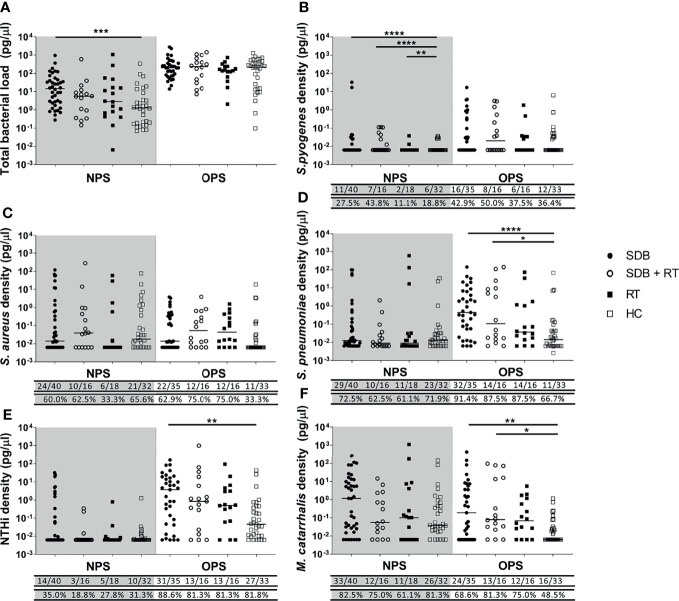
Total bacterial load **(A)** and specific bacterial density **(B–F)** in the nasopharyngeal swabs (NPS) and oropharyngeal swabs (OPS) from children with SDB (•), SDB+RT (⚬), RT (▪) and healthy children (HC) (▫). p value * < 0.05; **< 0.01; *** < 0.001; **** < 0.0001. Numbers underneath each graph represent the proportion of children in each group considered positive for each bacterial species with numbers on the top row and the corresponding proportion in the bottom. Samples not available: 1 NPS from a child with SDB+RT and 1 NPS from a healthy child; 5 OPS from children with SDB, 1 OPS from a child with SDB+RT and 2 OPS from children with RT.

*S. pyogenes*, *S. aureus, S. pneumoniae*, NTHi and *M. catarrhalis* were detected in nasopharyngeal and oropharyngeal swabs from children with adenotonsillar diseases as well as healthy controls. *S. pyogenes* was detected at relatively low frequencies in nasopharyngeal swabs compared to the other species, with only 2 out of 18 children diagnosed with RT (11.1%) positive for *S. pyogenes* in their nasopharynx compared to 27.5% of children with SDB, 43.8% of children with SDB+RT, and 18.8% of healthy children. *S. pyogenes* was detected more often in oropharyngeal swabs than nasopharyngeal swabs, with similar frequency of detection across all groups. *S. aureus* was detected more frequently in nasopharyngeal swabs from children with SDB, SDB+RT and healthy children compared to those with RT alone although this was not statistically different (60%, 62.5%, 65.6% and 33.3% respectively; p=0.143). In oropharyngeal swabs, *S. aureus* was detected more often from children with SDB (62.9%), SDB+RT (75%) and RT (75%) compared to healthy children (33.3%), (p=0.007). *S. pneumoniae* was frequently detected in nasopharyngeal swabs (>61%) and frequency did not differ between groups including healthy children. In contrast, *S. pneumoniae* was detected more frequently in oropharyngeal swabs from children with SDB, SDB+RT and RT compared to healthy children (91.4%, 87.5%, 87.5% and 66.7% respectively; p=0.046). NTHi was also frequently detected in oropharyngeal swabs, with more than 81% of children in each group including healthy controls being positive. In the nasopharynx, NTHi was detected in 19% to 35% of children with similar frequencies between groups including healthy children. *M. catarrhalis* was frequently detected in nasopharyngeal swabs, with over 60% of children positive. Similarly, this pathogen was detected in over 48% of oropharyngeal swabs, with a similar detection frequency across groups (**Figure 1**).

### Species-Specific Bacterial Densities in Oropharyngeal Swabs Were Lower in Healthy Children

Children with SDB alone had higher total bacterial loads in their nasopharyngeal swabs when compared to healthy children (p=0.001). While total bacterial loads in the nasopharynx were similar in children with SDB+RT, RT and healthy children. Total bacterial loads in the oropharynx were higher than in the nasopharynx and were similar between groups (**Figure 1A**).

The median density of *S. pyogenes* in nasopharyngeal swabs was higher in children with adenotonsillar diseases compared to healthy controls (SDB vs healthy p<0.0001; SDB+RT vs healthy p<0.0001; RT vs healthy p=0.003) (**Figure 1B**). While the median densities of *S. aureus, S. pneumoniae*, NTHi and *M. catarrhalis* in nasopharyngeal swabs were similar across groups (**Figures 1C**–**F**). In oropharyngeal swabs, the median densities of *S. pyogenes* and *S. aureus* were similar across all groups (**Figures 1B, C**). The density of *S. pneumoniae* in oropharyngeal swabs was higher in children with SDB and SDB+RT compared to healthy children (p<0.0001 and p=0.034 respectively) (**Figure 1D**). NTHi densities were also higher in children with SDB compared to healthy controls (p=0.002) but similar between other groups (**Figure 1E**). The densities of *M. catarrhalis* were higher in children with SDB and SDB+RT compared to healthy controls (p=0.01 and p=0.02 respectively) (**Figure 1F**).

### NTHi Was the Most Frequently Detected Pathogen in Tonsils From Children With Adenotonsillar Diseases

**Figure 2:**
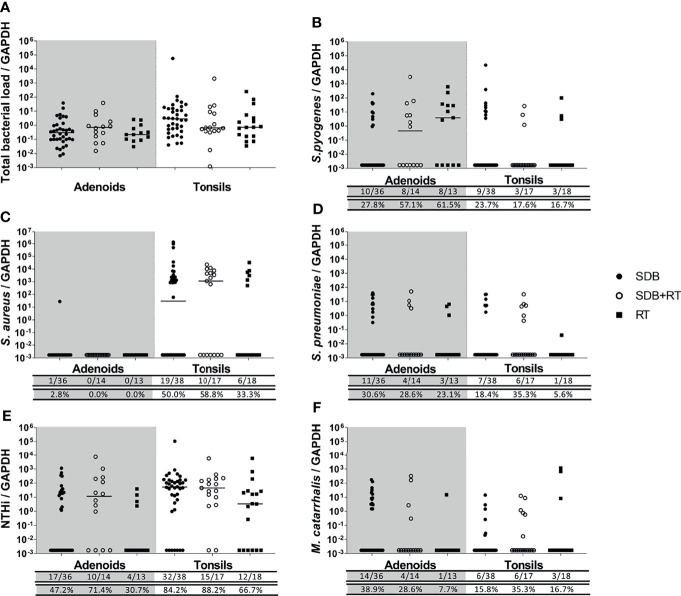
Total bacterial load **(A)** and specific bacterial density **(B–F)** in adenoids and tonsils from children with SDB (•), SDB+RT (⚬), RT (▪). Numbers underneath each graph represent the number of children in each group considered positive for each bacterial species ( top row) and the corresponding proportion (bottom row). Samples not available: 4 adenoids from children with SDB, 3 adenoids from children with SDB+RT and 5 adenoids from children with RT; 2 tonsils from children with SDB.

*S. pyogenes*, *S. pneumoniae*, NTHi and *M. catarrhalis* were detected in both adenoid and tonsil tissue from children with adenotonsillar diseases. NTHi was the predominant species detected in adenoid and tonsil tissue, with detection rates above 47% in adenoids and above 84% in tonsils from children with SDB and SDB+RT. The frequency of NTHi detection was lower in adenoids and tonsils from children with RT (30% and 66% respectively) but this was not significantly different when compared to other disease groups (**Figure 2E**). The frequency of *S. pyogenes* detection in adenoids was higher in children with RT compared to those with SDB (p=0.03). However, this was not the case in tonsil tissue with similar detection frequency of *S. pyogenes* across all disease groups (**Figure 2B**). *S. aureus* was also commonly detected in tonsil tissue from all children with adenotonsillar disease (33 to 58%), but was only present in the adenoid tissue from one child (**Figure 2C**). *S. pneumoniae* detection frequency in adenoids and tonsils was also similar across groups (23-30%), although only 1 child with RT had detectable *S. pneumoniae* in their tonsil tissue. The frequency of *M. catarrhalis* detection in adenoids was higher in children with SDB compared to RT (p=0.036) while the frequency of detection in tonsils was similar across groups (**Figure 2F**).

### Children Had Similar Total Bacterial and Species-Specific Densities in Their Adenotonsillar Tissue Regardless of Clinical Diagnosis

Total bacterial loads in adenoids and tonsils were similar between all children with adenotonsillar disease regardless of clinical diagnosis (**Figure 2A**). The densities of specific bacterial species were similar in adenoids and tonsils from children in all groups (**Figures 2B**–**F**). The density of *S. pyogenes* in adenoids tended to be higher in children with RT although this was not significant (p=0.054) (**Figure 2B**). *S. aureus* was found at high densities and at similar levels across groups in tonsils but was rarely detected in adenoids (**Figure 2C**). Densities of *S. pneumoniae* were comparable between groups in both adenoid and tonsil tissue (**Figure 2D**). NTHi was also present at similar densities in adenoids and tonsils across groups (**Figure 2E**). Densities of *M. catarrhalis* were low and comparable between groups in both adenoid and tonsil tissue (**Figure 2F**).

### Polymicrobial Detection Was More Common in Nasopharyngeal and Oropharyngeal Swabs Than Adenotonsillar Tissue

Concurrent detection of 3 or more of the 5 respiratory pathogens tested was common in nasopharyngeal and oropharyngeal swabs from children across all groups. Although, in oropharyngeal swabs, concurrent detection of 3 or more of the 5 respiratory pathogens occurred more frequently in children with adenotonsillar disease compared to healthy children (80% versus 57%, p=0.043). Adenoid and tonsillar tissues, only taken from children with adenotonsillar disease, were more likely to have only 1 or 2 species detected. The frequency of detection was similar across the 3 adenotonsillar groups (**Supplementary Table 2**).

When relative abundance of each species was compared, nasopharyngeal and oropharyngeal swabs exhibited high densities and diversity of detected species, although overall densities were lower in healthy children compared to those with adenotonsillar diseases (**Supplementary Figure 1**). Less polymicrobial interactions were observed in the adenoid and tonsillar tissues where usually only 1 or 2 species were present (**Supplementary Figure 2**).

### Tonsillar Polymicrobial Biofilms Are Also Evident in Children With SDB

**Figure 3:**
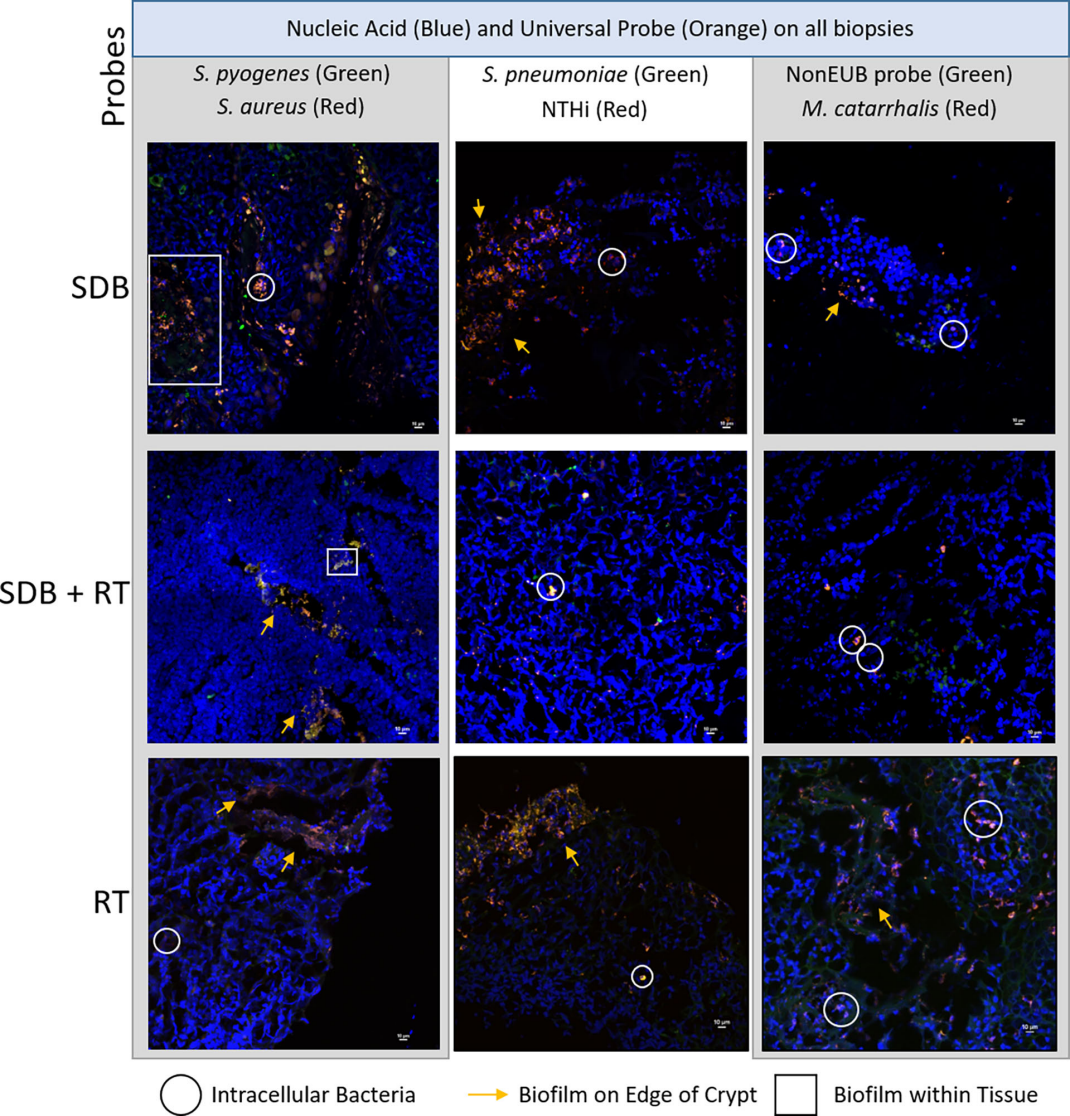
Representative maximum projections of intracellular bacteria, biofilms on surfaces and within tonsillar tissue from children with SDB, SDB+RT and RT. Scale bars represent 10µM.

Polymicrobial biofilms were detected in the tonsil crypts and on the tonsillar surfaces of tissue irrespective of clinical diagnosis (**Figure 3**). Biofilm presence, location and detection of intracellular bacteria were similar between the 3 disease groups (**Supplementary Table 3**). Species-specific probes identified the same bacterial species as were detected by qPCR with the universal bacterial probe confirming that other unidentified bacterial species were also abundant in biofilms. Some bacterial species were observed using FISH that were not detected using qPCR.

## Discussion

This study demonstrates the presence of 5 common respiratory pathogens in the upper respiratory tract and adenotonsillar tissues from children with adenotonsillar diseases. We observed that these 5 bacterial species are present just as often and at similar densities in children with SDB (including OSA) when compared to children diagnosed with RT, and in those with concurrent diagnoses of SDB and RT. Moreover, we have demonstrated surface and crypt-associated polymicrobial biofilms and intracellular bacterial pods in tonsil tissue from a subset of children presenting with each of these adenotonsillar diseases. Together, these data demonstrate the presence of bacteria in high densities and in formations that may contribute to the chronicity of infection in patients, including those presenting with SDB. Traditionally, children with SDB are considered to have disease of non-infectious origins in comparison to children diagnosed with RT, and have even been used as healthy comparators to children with RT in previous reports (Subtil et al., 2017; Dan et al., 2019). Our study challenges this dogma, and instead proposes that that the broad range of clinical presentations in children with SDB and RT may actually have a shared microbial aetiology. Our data also suggest that it is unlikely that a single bacterial species drives either an obstructive sleep phenotype or recurrent tonsillitis. We also showed that nasopharyngeal and oropharyngeal swabs, which have greater diversity of detected species, are not representative of the microbial profile of adenoids and tonsil tissues demonstrating that these swabs cannot be used as surrogates to determine what is happening in adenoids and tonsils leading to clinical disease. While these findings are contrary to our original hypothesis that children with SDB would have reduced bacterial loads and a lack of biofilms compared to those with RT, our data demonstrate an infectious phenotype associated with SDB and a high prevalence of NTHi and *S. aureus* infection. Importantly, these pathogens were demonstrated to be present within biofilms regardless of diagnoses which likely contributes to the chronicity of infection. The frequent and dense detection of NTHi and *S. aureus* in tonsillar tissue from our pediatric samples are consistent with previous studies. In tonsils removed from children and adults with OSA or RT, *H. influenzae* and *S. aureus* were the most frequently isolated species (particularly in children), implicating these species as major pathogens in adenotonsillar diseases. The authors also showed that biofilms were associated with clinical symptoms of tonsillar diseases such as apnoea and sore throat, suggesting that the recalcitrant nature of these diseases is due to microbial persistence (Alasil et al., 2013). In another study, which compared groups of children with RT to those with OSA, *S. aureus* was more frequently identified than *S. pyogenes* in tonsil tissue and at similar frequencies between the groups (Radcliff et al., 2019). This further supports the evidence that *S. pyogenes* is neither the sole nor major pathogenic determinant driving adenotonsillar disease and has implications for the clinical management of children with adenotonsillar disease. We recommend the investigation into the use of anti-biofilm agents or perhaps even NTHi and/or *S. aureus* targeted therapies while children with adenotonsillar disease are awaiting surgery or even before they reach this severe end of the disease spectrum.

Microbial involvement in SDB is further supported by recent studies. Bacterial 16S RNA sequencing revealed that the microbial profiles of adenoid and tonsil tissues in children with clinical symptoms of SDB were similar to children with RT, with frequent detection of *Streptococcus*, *Haemophilus*, *Moraxella*, *Neiserria* and *Staphylococcus* species and an overall dominance of *Haemophilus* (Johnston et al., 2019) Given that we and others have shown that the same bacterial species are involved in the pathogenesis of SDB as well as RT, host responses to these pathogens also need to be considered to explain the clinical presentations specific to each diagnosis. There are limited data on this in the context of adenotonsillar diseases, but it has been demonstrated that *S. aureus* isolates from tonsils from children with OSA or RT had mitogenic capacity, and that *S. aureus* presence was associated with elevated peripheral blood and tonsillar mucosal-associated invariant T (MAIT) cells, likely responding to the bacterially-derived superantigens. Interestingly, the proportion of tonsillar MAIT cells were reduced in children with RT compared to OSA but there were no differences between other CD4+ and CD8+ T cell compartments (Radcliff et al., 2019). These data suggest that there may be differences in immune development that influences disease pathogenesis, which should be investigated in future studies.

While the development of vaccines targeting NTHi and *S. aureus* may be important for the treatment/prevention of pediatric respiratory diseases, including adenotonsillar diseases, anti-biofilm agents may also be required. The identification of polymicrobial biofilms on tonsillar surfaces, in crypts and intracellularly in the small sub-group assessed in our study suggests that these persistence mechanisms contribute to the chronicity of infection in these clinical conditions. It may be pertinent in the future to investigate the potential use of topical anti-biofilm treatments (Brockson et al., 2014; Novotny et al., 2020), bacteriophage therapy (Kornienko et al., 2020), or vaccines targeting important proteins involved in biofilm formation and maintenance (Mokrzan et al., 2018; Novotny et al., 2019), to both prevent and treat these conditions, thus reducing the need for long term antibiotics or surgical intervention.

## Limitations

In Australia, due to considerable wait times for polysomnography versus adenotonsillectomy surgery, not all children with physician diagnosed SDB underwent polysomnography to confirm OSA. As a result only 23% of children with SDB had confirmatory polysomnography in our study and thus we used the validated TuCASA questionnaire (Goodwin et al., 2003) as a secondary tool to verify the SDB diagnosis. We acknowledge that the small group sizes in our study may not be representative of the complete spectrum of pediatric adenotonsillar diseases. However, our groups sizes are comparable (if not, slightly larger) to other studies investigating the aetiology and pathogenesis of adenotonsillar diseases. Furthermore, unlike other studies, we included groups of children to represent the breadth of adenotonsillar disease diagnoses, enabling assessment of microbial profiles that may have been unique to each. By using a targeted PCR approach focusing on the 5 major respiratory pathogens, we may have missed important microbes present in the tonsillar microbiota, but this was safe-guarded by using a universal bacterial qPCR and universal bacterial FISH probe to detect all bacteria present (Johnston et al., 2019). Future studies should include non-targeted profiling in conjunction with quantitative species-specific assays.

## Conclusions and Recommendations for Development of Future Therapeutics

We have shown that a similar polymicrobial infectious phenotype exists in children with SDB, RT alone, or with concurrent diagnoses of SDB and RT. Differences in clinical presentations are likely driven by host responses. The microbial profiles of the nasopharynx and oropharynx are not representative of the adenoids and tonsils. We identified NTHi and *S. aureus* as species to target and suggest that antimicrobial (including anti-biofilm) approaches may be relevant for treating children with SDB and/or RT. Vaccines targeting these pathogens may also be useful in reducing the overall prevalence of these diseases in pediatric populations.

## Data Availability Statement

The raw data supporting the conclusions of this article will be made available by the authors, without undue reservation.

## Ethics Statement

The studies involving human participants were reviewed and approved by Princess Margaret Hospital for Children Human Research Ethics Committee, Perth, Australia (1046/EP)(1385/EP). Written informed consent to participate in this study was provided by the participants’ legal guardian/next of kin.

## Author Contributions

RT had full access to all of the data in the study and takes responsibility for the integrity of the data and the accuracy of the data analysis. Concept and design: TM, RT, PR, LK, SV, KP, and HC. Acquisition, analysis or interpretation of data: all authors. Drafting of the manuscript: ES, RT, PR, and LK. Critical revision of the manuscript for important intellectual content: all authors. Statistical analysis: ES. Administrative, technical or material support: TM, SV, KP, and HC. Supervision: RT, PR, and LK. All authors contributed to the article and approved the submitted version.

## Funding

This study was funded by the Western Australia Department of Health Telethon Perth Children’s Hospital Research Fund. TM was a recipient of a University of Western Australia International Postgraduate Fellowship and Stan and Jean Perron top-up scholarship. LK was a recipient of an Australian National Health and Medical Research Council (NHMRC) Career Development Fellowship (#1061428). RT was a recipient of a BrightSpark postdoctoral fellowship.

## Conflict of Interest

The authors declare that the research was conducted in the absence of any commercial or financial relationships that could be construed as a potential conflict of interest.

## Publisher’s Note

All claims expressed in this article are solely those of the authors and do not necessarily represent those of their affiliated organizations, or those of the publisher, the editors and the reviewers. Any product that may be evaluated in this article, or claim that may be made by its manufacturer, is not guaranteed or endorsed by the publisher.
