# Supplementary figures and images for: Sleep Disordered Breathing and Recurrent Tonsillitis Are Associated With Polymicrobial Bacterial Biofilm Infections Suggesting a Role for Anti-Biofilm Therapies
Source: Front Cell Infect Microbiol. 2022 Feb 28;12:831887. doi: 10.3389/fcimb.2022.831887 (PMC8918577; doi:10.3389/fcimb.2022.831887)

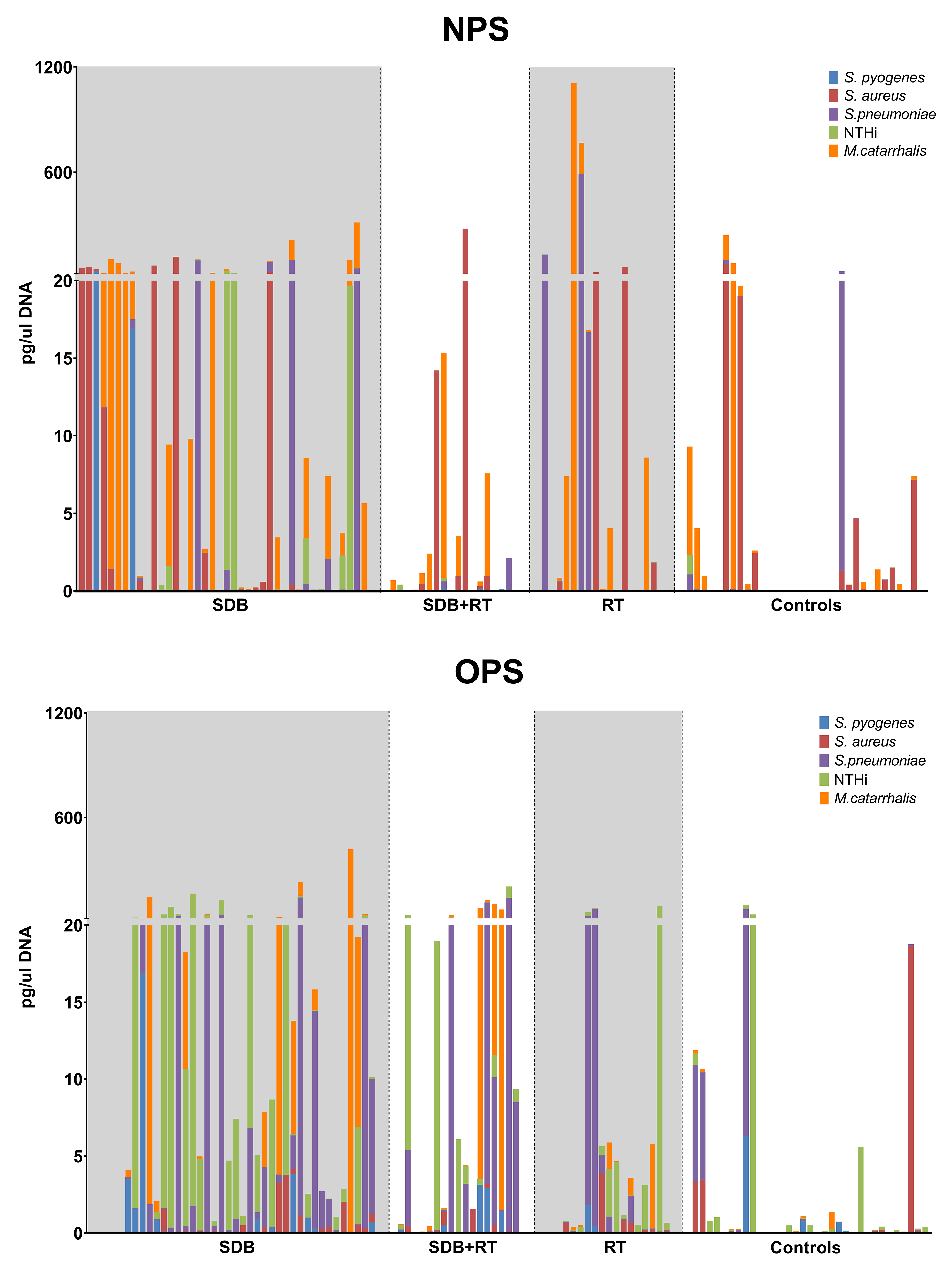

Supplement: Supplementary file 1 [file Image_1.tif]

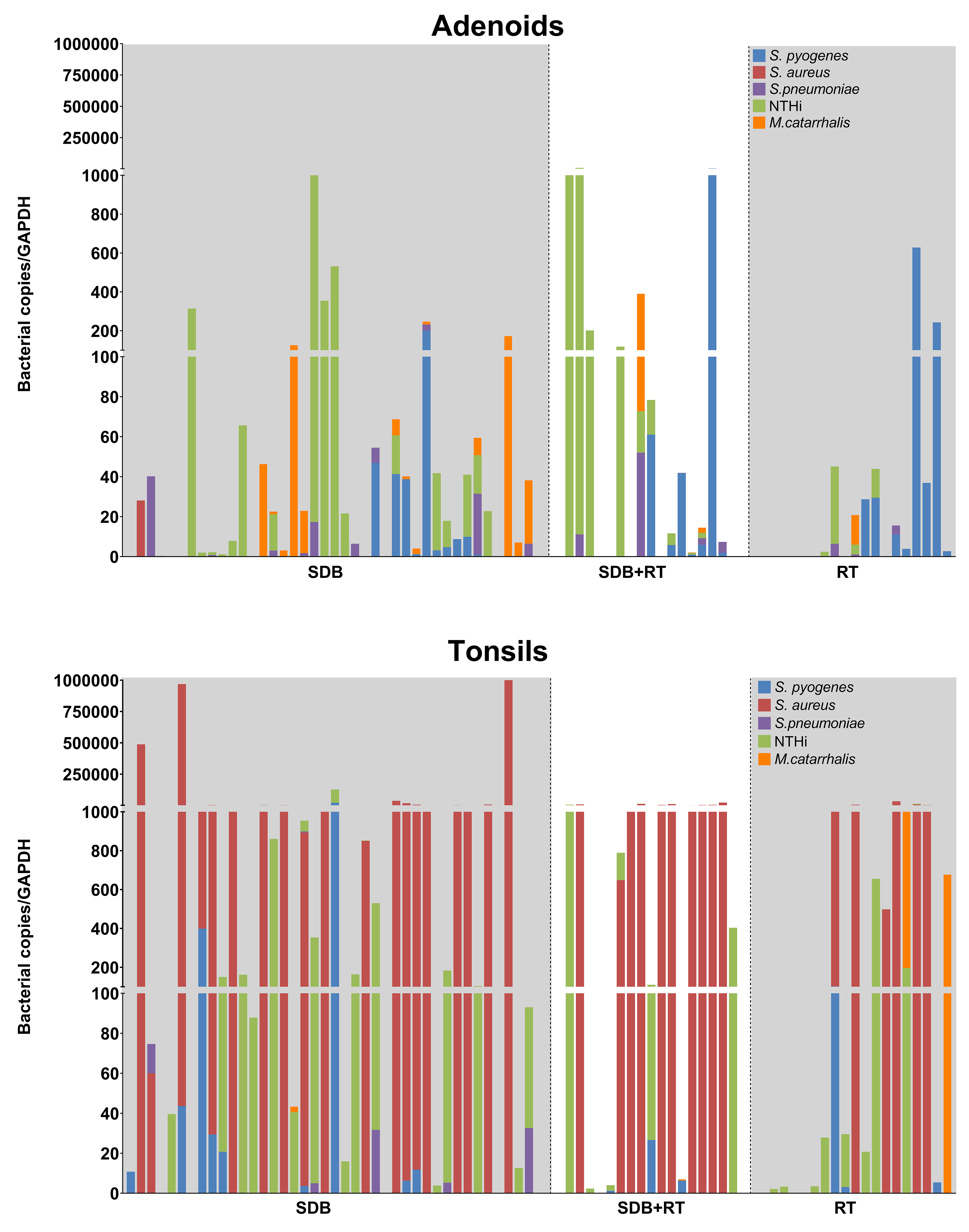

Supplement: Supplementary file 2 [file Image_2.tif]
