# Supplementary material for: Sleep Disordered Breathing and Recurrent Tonsillitis Are Associated With Polymicrobial Bacterial Biofilm Infections Suggesting a Role for Anti-Biofilm Therapies
Source: Front Cell Infect Microbiol. 2022 Feb 28;12:831887. doi: 10.3389/fcimb.2022.831887 (PMC8918577; doi:10.3389/fcimb.2022.831887)
Supplement: Supplementary file 3 [file Table_1.docx]

**Sleep Disordered Breathing and Recurrent Tonsilitis are associated with polymicrobial bacterial biofilm infections suggesting a role for antibiofilm therapies**

**Supplementary Material**

**Supplementary Table 1. 16S rRNA probes used for fluorescent *in situ* hybridisation**

| **Probe Name** | **Target bacterial species** | **Sequence** | **Fluorescent label** | **Formamide concentration** |
| --- | --- | --- | --- | --- |
| EUB338 | Universal bacteria | GCT GCC TCC CGT AGG AGT | AF 546 | 20%, 5% |
| GAS | *S. pyogenes* | TTC CAA AGC GTA CAT TGG TT | AF 546 | 20% |
| Haeinf | *H. influenzae* | CCG CAC TTT CAT CTT CCG | AF 633 | 20% |
| Mrc88 | *M. catarrhalis* | CCG CCA CUA AGU AUC AGA | AF 633 | 5% |
| NON EUB | Nonsense -ve control | ACT CCT ACG GGA GGC AGC | AF 488 | 5% |
| Saur | *S. aureus* | GAA GCA AGC TTC TCG TCC G | AF 488 | 20% |
| Spn | *S. pneumoniae* | GTG ATG CAA GTG CAC CTT | AF 488 | 20% |

**Supplementary Table 2. Frequency of co-detection for bacterial species**

|  | SDB  n (%) | SDB+RT  n (%) | RT  n (%) | Healthy  n (%) | p value |
| --- | --- | --- | --- | --- | --- |
| **NPS** |  |  |  |  |  |
| 1 | 4 (10) | 2 (12.5) | 4 (22.2) | 6 (18.8) | 0.585 |
| 2 | 10 (25) | 4 (25) | 3 (16.7) | 6 (18.8) | 0.852 |
| ≥3 | 26 (65) | 10 (62.5) | 8 (44.4) | 20 (62.5) | 0.502 |
| **OPS** |  |  |  |  |  |
| 1 | 0 | 0 | 1 (6.3) | 5 (15.2) | 0.043 |
| 2 | 5 (14.3) | 3 (18.8) | 2 (12.5) | 9 (27.3) | 0.491 |
| ≥3 | 30 (85.7) | 13 (81.3) | 13 (81.3) | 19 (57.6) | 0.043 |
| **Adenoids** |  |  |  |  |  |
| 1 | 19 (52.8) | 4 (28.6) | 7 (53.8) | N/A | 0.269 |
| 2 | 9 (25) | 4 (28.6) | 3 (23.1) | N/A | 0.944 |
| ≥3 | 5 (13.9) | 4 (28.6) | 1 (3.2) | N/A | 0.294 |
| **Tonsils** |  |  |  |  |  |
| 1 | 11 (28.9) | 3 (17.6) | 9 (50) | N/A | 0.106 |
| 2 | 16 (42.1) | 5 (29.4) | 5 (27.7) | N/A | 0.481 |
| ≥3 | 9 (23.7) | 8 (47.1) | 2 (11) | N/A | 0.047 |

NPS= nasopharyngeal swab; OPS = oropharyngeal swab

**Supplementary Table 3. Polymicrobial biofilms in tonsil tissue**

|  | S. pyogenes | | *S. aureus* | | *S. pneumoniae* | | NTHi | | *M. catarrhalis* | | Intracellular  Bacteria* | Biofilm present |
| --- | --- | --- | --- | --- | --- | --- | --- | --- | --- | --- | --- | --- |
|  | PCR | FISH | PCR | FISH | PCR | FISH | PCR | FISH | PCR | FISH |  |  |
| SDB | 3/3 | 2/3 | 3/3 | 3/3 | 1/3 | 2/3 | 3/3 | 3/3 | 1/3 | 3/3 | 3/3 | 3/3 |
| SDB +RT | 0/3 | 1/3 | 3/3 | 3/3 | 2/3 | 2/3 | 3/3 | 3/3 | 3/3 | 3/3 | 3/3 | 3/3 |
| RT | 1/3 | 1/3 | 3/3 | 3/3 | 0/3 | 1/3 | 2/3 | 3/3 | 1/3 | 3/3 | 3/3 | 3/3 |

FISH was performed on tonsils from 3 children in each group. The positive identification of specific bacterial species detected by either qPCR or FISH in each sample was noted as x/3.

**Supplementary Figure 1:** Relative abundance of each species in nasopharyngeal swabs (NPS) and oropharyngeal swabs (OPS). Each column represents an individual child, and each colour represents a specific species according to the amount detected.

**Supplementary Figure 2:** Relative abundance of each species in adenoids and tonsils. Each column represents an individual child, and each colour represents a specific species according to the number of bacterial copies detected.
